# Plasticity of Adult Sensorimotor System in Severe Brain Infarcts: Challenges and Opportunities

**DOI:** 10.1155/2012/970136

**Published:** 2012-03-22

**Authors:** Annette Sterr, Adriana Bastos Conforto

**Affiliations:** ^1^School of Psychology, University of Surrey, Guildford GU2 7XH, UK; ^2^Neurology Clincal Division, Hospital das Clinicas, São Paulo University, 05403-000 São Paulo, SP, Brazil; ^3^Instituto Israelita de Ensino e Pesquisa, Hospital Israelita Albert Einstein, 05652-900 São Paulo, SP, Brazil

## Abstract

Functional reorganization forms the critical mechanism for the recovery of function after brain damage. These processes are driven by inherent changes within the central nervous system (CNS) triggered by the insult and further depend on the neural input the recovering system is processing. Therefore these processes interact with not only the interventions a patient receives, but also the activities and behaviors a patient engages in. In recent years, a wide range of research programs has addressed the association between functional reorganization and the spontaneous and treatment-induced recovery. The bulk of this work has focused on upper-limb and hand function, and today there are new treatments available that capitalize on the neuroplasticity of the brain. However, this is only true for patients with mild to moderated impairments; for those with very limited hand function, the basic understanding is much poorer and directly translates into limited treatment opportunities for these patients. The present paper aims to highlight the knowledge gap on severe stroke with a brief summary of the literature followed by a discussion of the challenges involved in the study and treatment of severe stroke and poor long-term outcome.

## 1. Background

The seminal discovery of adult brain plasticity in animals and humans has hugely influenced the theories and concepts applied in neurorehabilitation research and their translation into practice, in particular with regards to movement deficits in acquired brain injury [1, 2]. This work directly translated into new and, in some cases, more efficient interventions for patients with mild to moderate hemiparesis (e.g., [3–5]). However, much less research has specifically investigated the reorganization of the motor system in patients with poor or minimal functional abilities and, most critically, the rehabilitation of these patients [6, 7]. This gap in the literature may be present for a number of reasons. Firstly, standard rehabilitation approaches are difficult to apply when patients have little voluntary movement. Secondly, the lack of funding for regular one-to-one physical therapy sessions beyond the postacute phase, together with the common assumption that substantial improvements in functional motor ability are unlikely once the first 6 months of recovery have passed, primes the health care system and patients to accept the status quo. While the latter is true for the whole range of functional recovery, the impact is particularly grave for patients with poor functional recovery and also affects the research effort. Thirdly, the motor system of patients with poor residual recovery cannot easily be studied with the paradigms adopted from basic science research, such as finger opposition or grip movements. These methodological challenges can be overcome by excluding patients with high levels of spasm or by limiting the study group to those with relatively good levels of motor control. As a result, the motor system of patients with very poor recovery, in particular in chronic state, has been studied much less than that of patients with mild or moderate impairment. This translates directly into studies on treatment efficacy, and indeed, the availability of suitable treatments per se.

Based on the aforementioned considerations we argue that research on the mechanisms of long-term recovery, and their interaction with treatment efficacy, needs to widen its focus to the population of stroke survivors with severe long-term motor deficits. Hereinafter we briefly summarise the present literature and further discuss the challenges involved in the study and treatment of patients with minimal motor recovery.

## 2. Animal Models of Focal Ischemia versus Human Strokes

On average, 80% of all strokes are ischemic, and 20% are hemorrhagic [8]. Upper limb paresis occurs in 85% of the patients and substantially impacts disability in the long term [9]. Animal models have tried to reproduce stroke lesions that occur in humans, with variable degrees of success. The majority of focal ischemia models involve the middle cerebral artery (MCA) territory, the most commonly affected arterial territory in ischemic strokes in humans [10].

### 2.1. Animal Models of Focal Ischemia

Rat focal ischemia models are frequently used because of low cost, similarities between vasculatures of rats and humans, fewer concerns from the general public compared to nonrodents, and availability of clear behavioral outcomes [11–13].

Severe MCA infarcts in rodents leading to long-lasting sensorimotor and cognitive deficits can be produced by proximal MCA occlusion induced by electrocoagulation. Complete or partial MCA occlusion can be also achieved by insertion of an intraluminal filament. In the filament model, mortality can be high if a large stroke is produced [14].

Other models lead to less severe morbidity and mortality. For example, if endothelin-1 [15], a vasoconstrictor drug, is injected topically or intracerebrally, the forelimb motor cortex is typically spared, but infarcts vary in location and extension depending on the sites and route of drug administration. In models of occlusion of the distal MCA or its branches, cortical frontoparietal infarcts are associated with less severe deficits than in the cortico-subcortical infarcts produced by proximal MCA occlusion [16]. Furthermore, multiple embolic infarcts can be produced in brain areas supplied by the MCA, after injection of microspheres [17], macrospheres [18], a thrombotic clot, or purified thrombin into the internal carotid artery [19, 20]. Finally, light activation of photosensitive dyes such as Rose Bengal makes small cortical infarcts possible, by occlusion of cortical vessels [21].

Many of these techniques have not been applied exclusively to small animals such as rats, mice, gerbils, or rabbits, but also to cats, dogs, pigs, and monkeys. Larger animals have proportions of gray/white matter that are more similar to those found in human brains, in contrast with lissencephalic brains of rats and mice [22]. However, technical limitations and costs limit widespread use of focal ischemia models in larger animals. It is recommended, for instance, that new drugs be tested first in rodents, before efficacy is investigated in gyrencephalic species [23].

Animal models are a double-edged sword when used to understand stroke pathogenesis. They offer powerful opportunities: the possibility to objectively monitor behavioral outcomes, manipulate experimental conditions, and obtain images (MRI, microPET) or intracortical recordings of neuronal activity in living animals; to scrutinize molecular mechanisms of cell death and recovery; to work with strains and transgenic animals that present hypertension, atherosclerosis and obesity, among other factors that are common in patients with stroke; and to perform postmortem histological evaluations.

Still, conclusions based on animal models of focal ischemia must be examined with caution. For instance, small, selective cortical infarcts leading to mild sensorimotor deficits that tend to improve quickly are present in some of the models but are not common in humans, even though similar clinical features can occur in subcortical, lacunar infarcts. In addition, background pathophysiology is not shared between focal cerebral ischemia in rodents and humans. Fast arterial recanalization is achieved in several animal models, while in humans recanalization can occur either after r-tPA administration or spontaneously but, unfortunately, does not happen at an early phase after ischemic injury in most patients [8, 24–27]. Lack of arterial recanalization is strongly associated with more severe strokes and lower probabilities of recovery. Furthermore, rodent models have often included young healthy animals while, in humans, the bulk of strokes is concentrated in the elderly [28–32].

Age is a potent predictor of poor outcome in humans [33, 34]. Interestingly, despite high mortality rates in old mice, levels of recovery at four weeks have been reported to be similar in aged and young animals [35]. Other factors may contribute to poor outcome in humans, ranging from different mechanisms underlying strokes in different age groups to self-fulfilling prophecies in stroke care in the aged, and importantly, to comorbidities that impact recovery. Diabetes mellitus and atrial fibrillation, for instance, are significantly associated with more severe outcomes and lower chances of recovery [36, 37].

Hypertensive rats have poorer collateral blow flow and also worse outcomes after focal ischemia than nonhypertensive animals [38]. The demand for stroke models in hypertensive, obese, and aged rodents has been underlined [22]. Contrasts between the compelling efficacy of a myriad of drugs in animal models of neuroprotection and the systematic failure of the same drugs when administered to patients in clinical trials underscore the requirement for animal models that more realistically approach human strokes [13, 22, 39]. However, bigger rates of complications and mortality in aged or unhealthy animals, technical difficulties (anesthesia, complex surgeries in less flexible arteries, etc.), and hence the greater costs involved present challenges for advancements of research in the field. In particular, high mortality rates limit the opportunity to study recovery of animals with severe strokes in the chronic phase.

In summary, there is a gap between pathogenesis and clinical features of severe strokes in humans and pathogenesis and clinical features of the most widely used models of focal ischemia in animals. High costs and lower expectations for substantial improvements in activity or quality of life are also major obstacles for rehabilitation of patients with severe strokes. Still, as stroke mortality decreases in parallel with advances in health care, it is expected that more patients with severe strokes will survive the acute phase over the next decades. At the moment, successful rehabilitation strategies for these patients are largely insufficient. Thus, animal models of focal ischemia that match severe human strokes more closely are deeply needed.

Despite these limitations, animal models have provided unique insight on plastic mechanisms underlying sensorimotor recovery after focal ischemia, and on how to enhance beneficial patterns of reorganization to obtain behavioral gains (e.g., [40]). Constraint-induced movement therapy, for instance, shown to improve motor outcomes in humans with different types and sizes of strokes, was developed based on seminal studies that underscored the importance of the amount of use of the paretic limb to promote enhancement of motor function [41, 42]. The phenomenon was observed in monkeys with small cortical infarcts submitted to intracortical microstimulation and behavioral testing [42]. As mentioned before, such small infarcts are rarely observed in humans, but still, the information gained by the model was a major step forward in stroke rehabilitation. Constraint-induced therapy has now been successfully applied to patients with various types of strokes, with more severe deficits and worse motor prognoses than the monkeys included in the model of forced use of the affected limb [1, 3, 43, 44].

### 2.2. Studying Recovery after Stroke in Humans: Neurophysiology and Neuroimaging

Over the past decades, neuroimaging and neurophysiology techniques have emerged as paramount tools to study brain reorganization in humans [45–50]. Overall, functional neuroimaging studies in patients with stroke have shown that good performance is associated with augmented activity in preexisting networks during a motor task, rather than assignment of networks that are not overtly active during these tasks in healthy subjects. Furthermore, incremental activity in undamaged areas may negatively impact motor performance.

For instance, according to the model of interhemispheric inhibition, an imbalance in activity between the ipsilesional and the contralesional primary motor cortex (M1) can occur after stroke [51, 52]. The ipsilesional M1 may be less able to inhibit the contralateral M1. The disinhibited contralesional M1, in turn, may excessively inhibit the ipsilesional M1. A number of studies have shown that this imbalance in activity between the two hemispheres can impair motor performance of the affected hand, at least in some patients in the chronic phase after stroke [51, 52]. Whether interhemispheric callosal fibers mediate this phenomenon or whether it depends on changes in activity of cerebellar or thalamic pathways, remains an open question [52]. However, the vast majority of published studies that investigated effects of modulation of interhemispheric inhibition only included patients with mild to moderate motor impairments, likely due to difficulties in developing tasks and applying available neurophysiology and neuroimaging tools to more severely affected patients [51–59].

In regard to recruitment of ipsilesional or contralesional secondary motor areas, striking differences in patterns of function were unveiled when paradigms of investigation were applied to patients with poor recovery, compared to those of less affected patients or healthy subjects. Recruitment of secondary motor areas occurs when the outflow from M1 is disconnected from the spinal cord in large cortical, cortico-subcortical or subcortical strokes, as well as in strokes that strategically damage the corticospinal tract [60, 61]. At least in part, more pronounced patterns of activity in secondary motor areas in the ipsilesional and/or contralesional areas are associated with more severe disruption of the corticospinal pathway, and excessive activation of secondary motor areas correlates with poorer motor behavior [48, 50, 62]. Whether excessive activations of secondary motor areas or contralesional M1 are maladaptive, whether they represent the best possible remodeling option after severe injury, or whether they may play “hero” or “villain” roles depending on motor tasks/circumstances remains to be determined.

There are indications that motor performance may actually rely on activity of contralesional areas in more severely affected patients. For instance, it has been shown that transient disruption of the contralesional dorsal premotor cortex by transcranial magnetic stimulation slows motor performance of the paretic hand to a greater extent in patients with worse motor performance compared to less impaired patients [63]. In addition, activation of the *contralesional *dorsal premotor cortex has been demonstrated to be greater in patients with more severe hand motor impairment, compared to healthy subjects and to less affected patients [63]. On the other hand, disruption of the *ipsilesional *dorsal premotor cortex increases reaction times in patients with chronic stroke and mild motor impairments [64].

Together, these studies provide evidence that (1) contralesional areas are positively, functionally relevant in at least some well-recovered patients in the chronic phase; (2) in patients with severe motor deficits, behavioral gains, albeit small, may occur by augmented activity of networks that are normally either minimally active or not active at all in healthy brains. Severity of motor impairment seems to be a key factor influencing patterns of rewiring after stroke, but age, brain status before stroke, intensity, and timing of rehabilitative interventions, among other factors, are also likely to play pivotal roles in the process [33, 34, 65–67].

A key concept to develop effective rehabilitation interventions is heterogeneity of mechanisms underlying stroke as well as plastic processes that lead to recovery of function after neuronal injury. As they say, “different strokes for different folks”. Stroke lesions and clinical presentations vary across patients. Mechanisms underlying neurological impairment, recovery of activity, and participation are also distinct.

Until now, most proof-of-principle studies or clinical trials have excluded patients with severe sensorimotor impairments and the lack of evidence-based effective interventions has nurtured a nihilistic approach for rehabilitation of these patients. Therefore, expansion of research about mechanisms underlying reorganization after severe strokes is imperative.

Moreover, patients with severe sensorimotor impairments often present with cognitive impairments, depression and are faced with massive changes in psychosocial interactions [68–70]. Further research efforts must not only address restoration of sensorimotor function but also incorporate an integrative approach to target neuropsychiatric domains, personal experience/expectations, environmental conditions, and psychosocial factors.

## 3. Capitalizing on Adult Brain Plasticity to Enhance Motor Recovery: Neural and Behavioral Considerations

Changes to the functional organization of neural representations and their behavioral concomitants have been described for a number of human study models such as amputates (e.g., [71, 72], musicians [73, 74], blind (e.g., [75–78]) and deaf persons (e.g., [79, 80]), as well as learning paradigms (e.g., [81–84]). Together these studies suggest that sensory representations are sensitive to enhanced or altered sensory stimulation. This knowledge has the prospect to devise interventions that capitalize on the plastic capacities of the adult brain, mainly training- or practice-based interventions. The mechanisms of brain plasticity interact with psychological processes and behavior and together provide a number of considerations for the conceptualization of interventions.

### 3.1. Injury-Induced Plasticity

Injury to the peripheral or central nervous system changes receptive field characteristics of neurons and neural representations through deprivation of the original afferent inputs these neurons receive [85, 86]. The key mechanisms driving this change are the disinhibition of silent synapses, and the loss of inhibitory or excitatory inputs from lesioned neural populations connected to none-lesioned regions, including homologous areas in the two hemispheres [87]. In both cases, it is important to appreciate the effects of functional changes in nonlesioned areas and their influence on the control of the impaired behavior. As a consequence, rehabilitation efforts should not only focus on the impaired function per se, for example, the affected upper-limb in the case of hemiplegia, but also consider the effects of use or nonuse of the lesser-affected extremities. This is particularly relevant for patients with poor recovery as they will most likely entirely rely on the less-affected extremity in everyday behavior. This might, at least in theory, increase the interhemispheric inhibition exerted by the nonlesioned hemisphere.

### 3.2. Use-Related Reorganization

Sensory stimulation and practice shapes neural representations [88]. These changes are most likely driven through Hebbian mechanisms as well as dendritic and axonal processes [89]. Critically, use-related reorganization is not driven by increased neural activation alone but heavily depends on the behavioral relevance of the activity [90–92]. This association of sensory stimulation and consequential changes in neural representations and receptive field parameters has been demonstrated most elegantly in monkeys who received auditory and tactile simulation within the same protocol. Behaviorally, the animals were only rewarded for responses in one of the two stimulation modalities, and subsequent changes in the brain were only observed for the rewarded modality. Thus this data indicates that neural representations are only susceptible to stimulation-induced reconfigurations if this stimulation is attended to and of behavioral significance. Therefore, we argue that interventions aiming to enhance recovery through the induction of practice-induced plasticity not only need to focus on the actual practice element of the intervention but also consider how the intervention characteristics enforce attention and provide tangible and motivating feedback.

### 3.3. Generic Effects of Information Processing Influencing Motor Cognition

Motor control is a complex behavior that goes far beyond motor execution and the processes typically associated with primary motor cortex function. Cognitive processes such as motor planning, error-monitoring and attention can heavily influence motor performance. Motor rehabilitation research, however, typically conceptualizes motor function as the ability to execute a movement with little consideration being given to the cognitive processes that might influence this ability. For example, few studies have investigated how much patients with hemiplegia benefit from advanced movement preparation. Anticipatory processes and motor planning modulate motor performance. The respective behavioral costs or benefits result from perceptual, cognitive, and motoric components of the stimulus-response cascade, such as stimulus-response mapping and response selection. Few studies have investigated advanced movement preparation in patients. A study by Verleger et al. [93] suggests that well-recovered patients show little difference to controls in a motor priming task. Using a similar paradigm in patients with poor recovery, our group found marked behavioural and electrophysiological difference between patients and matched control. Most strikingly, the data suggests that patients are more sensitive to advance information (manuscript submitted). Thus, it appears that visual precues can facilitate or hinder apparent affected arm abilities, and that the magnitude of this effect is modulated by the severity of the motor deficit. While more research is needed to fully understand the interaction of cognitive processes, stroke severity, and motor performance, the findings summarised previously suggest that using advance movement information in a cognizant and explicit manner may be a beneficial addition to rehabilitation interventions.

### 3.4. Behavior Modification

In order to translate improvements in motor ability into real-world benefits, treatments have to obtain not only better motor control over the affected arm, but also a transfer of these newly acquired abilities into the curriculum of everyday behaviors. This translation essentially requires a modification of behavior. Behavior change can be facilitated through a number of measures falling under the CBT (cognitive behavioral therapy) umbrella. These measures use not only learning principles, which in themselves are likely to facilitate use-related plasticity processes, but also tools that enhance motivation, adherence, and coping. The latter, again, are likely to increase the patient's engagement with the intervention, which in turn is likely to improve outcome. Conceptualizing the rehabilitation of motor function as changing motor behavior rather than improving motor ability represents a significant shift in theoretical perspective. It implies that practice-based interventions should include treatment strategies that actively support sustained learning and behavior change, through the explicit use of learning principles and CBT elements. The latter facilitates not only real-world benefit but also enhances the processes of use-related functional reorganization that drive improvements in motor control.

### 3.5. Psychological Barriers

Interview data (unpublished) from patients with severe chronic hemiplegia indicates that these patients experience disproportionate psychological and service-related barriers. For example, statements such as “I have lost my hand,” “I hate it (the hand) and want to make it invisible”, and “it (the hand) looks horrible and is no good; I'd cut it off if I could” suggest that patients do not only face the actual physical impairment but also face psychological barriers to using any residual ability. This aspect is particularly important for interventions that rely on motor practice because the patient's engagement with the intervention is directly linked to their ability to cope with their disability. However, more often than not physiotherapy is provided without considering the psychological barriers associated with the use of the hemiplegic hand. We therefore argue that in order to improve the prospects for patients with low-functioning hemiparesis it is necessary to better understand the psychology of poor recovery and build that knowledge into motor rehabilitation interventions.

In addition to the psychological barriers low-functioning patients seem to experience, our interview data suggests a strong perception amongst these patients that the care system is presumptuous and does not provide for them. This is illustrated by comments such as “my doctors gave up on me after 3 month,” “my GP said that my hand will not get better,” “my physio tried to work with my hand initially but soon gave up,” “the OT only taught me how to manage things (with my good hand), I guess this was because she knew that my (paretic) hand would be no good,” and “there is nothing they can do for me because I cannot move.” These comments highlight not only the need to understand the patient perspective and a holistic approach to long-term care of these patients, but also a critical need to develop motor rehabilitation treatments that help patients to enhance their residual motor ability and enhance its real world benefit.

### 3.6. Tiredness, Fatigue, and Daytime Sleepiness

Tiredness and fatigue are a common concern in patients after stroke. This affects not only the patients' general levels of activity but, of course, also the level of engagement with the therapy process. Not a lot can be gained in a therapy session with a tired patient! Moreover, tiredness and fatigue are linked to sleep, and sleep is likely to be a modulating factor of recovery. For example, Terdouzi et al. [94] has shown that poorer sleep is associated with poorer long-term outcome. Moreover, patients with chronic low-functioning hemiparesis seem to suffer from sleep difficulties at least as frequently as the general population [95]. This is an important point for a number of reasons. Firstly, poor sleep is often associated with higher daytime sleepiness. As a consequence, patients may be more sleepy and hence less active during the day [96]. Secondly, poor sleep negatively affects daytime performance and information processing. It is therefore likely to further aggravate the difficulties patients already have with activities of daily living and to reduce the benefit patients can get from therapy and other activities. Finally, an increasing body of literature suggests that sleep enhances brain plasticity in general and procedural learning specifically (e.g., [97–100]). Assuming that brain plasticity is the main driver for the recovery of function, good and sufficient sleep is likely to facilitate and probably enhance the rehabilitation effort. Support for this assumption is provided by a series of studies suggesting that motor learning can be positively influenced by sleep [101, 102].

The issues raised previously are relevant to all patients but probably have greater significance in patients with poorer recovery. Therefore, treatments for these patients in particular should incorporate measures to counter tiredness and fatigue and monitor the quality of nocturnal sleep as well as daytime sleepiness.

## 4. Overt and Covert Movement: Alternative Ways of Stimulating the Motor System

Jeannerod's theory of neural simulation [103] suggests a shared neural network for the control of overt and covert movement modalities such as movement observation and motor imagery. This theoretical framework provides a powerful tool for research in patients with poor recovery. 

Generally, evidence from behavioral, neuroimaging, and psychophysiological studies confirms Jeannerod's notion of equivalence in healthy populations. For example, several fMRI studies have shown comparable activations in the cortical motor regions when participants observe or imagine hand movements (e.g., [104–107]). Thereby, these activities are similar to the activations obtained when the same movements are actually performed in the scanner. Typically, these studies use tasks that can be practiced prior to scanning and can be performed in the scanner. While this approach yields interesting and important insights, it lacks ecological validity, particularly with regard to the rehabilitation context. Taking these considerations on board, Szameitat and colleagues [108, 109] studied motor imagery of complex actions, such as eating with knife and fork or running. Using an fMRI paradigm, they successfully demonstrated that imagery of such complex movements is feasible in the scanner environment and leads to meaningful activations in the motor system. These findings are not trivial because complex everyday actions cannot be practiced prior to the scanning. The person will therefore rely on their motor memory rather than the experience obtained through practicing the actual task (e.g., finger tapping) immediately before the scanning begins. In this sense, the imagery of complex movements, by default, affords less stringent experiment control. However, if motor imagery is to be used as a study tool for patients with poor recovery, their inability to move will prevent practice prior to scanning, and therefore, these patients will also perform the task by relying on their motor memory. Evidence showing the feasibility and suitability of a paradigm that omits practice is therefore particularly relevant for the application in patients with poor residual recovery. Moreover, the study of complex actions with fMRI opens the door to the investigation of the representation of activities of daily living and the alteration of those representations throughout recovery and/or treatment.

As mentioned previously, the cognitive processes involved in advance movement preparation are likely to play an important part in the person's ability to function in everyday life but might also enhance or hinder the rehabilitation effort. Understanding to what extend the principle of equivalence also holds for the higher cognitive processes involved in motor planning is therefore important. In a series of EEG experiments, Kranczioch and colleagues [110, 111] directly compared execution, imagination, and observation of finger movements in an advanced motor preparation paradigm. These studies firstly showed that the EEG-derived ERPs provide a good tool for the study of covert movements. This is important because the high temporal resolution of EEG typically requires precisely timed stimuli, which is a challenge for the imagery condition. At the same time, not all patients can part-take in MRI scanning (e.g., because of metal in their body) and EEG can therefore provide an alternative method to study motor processes.

Covert movements not only provide a good vehicle to study the reorganized motor system of those patients unable to execute the kind of controlled hand movements used in experimental paradigms requiring overt responses, but can also be employed to induce enhanced neural activation of motor circuitries which aids functional reorganization and recovery. Coined by Sharma and colleagues as “a backdoor to the motor system after stroke” [112], therapeutic approaches using covert movement modalities have recently been tested [7, 113–116]. While initial evidence is promising, there are a number of questions that need to be answered in due course. For example, literally all our knowledge on covert movement has been obtained in younger persons, typically University students. Aging is known to affect the motor system (e.g., [117–119]); in fact, the way a person moves is quite indicative of older age and frailty. It is therefore not inconceivable that motor-specific mechanisms of covert movement, determined in younger populations, are differentially affected by age. Similarly, the ability to imagine, or to focus on stimuli during observation, relies heavily on cognitive and perceptual processes [120] and may therefore, again, be modulated by age. The latter may of course also be affected by the stroke [121, 122]. More research that characterizes covert movement in healthy older persons is therefore needed to help tailor treatment development. In addition, it is presently unclear whether imagery and observation provide equally suitable treatment pathways, and how this interacts with lesion location. While several studies have explored the effects of mental practice on recovery [115], there is, to the best of our knowledge, no direct comparison between these methods. Pilot data from our group [123] suggests that motor imagery is best able to activate the reorganized motor system in patients with chronic sever hemiparesis.

## 5. Concluding Remarks

Poor long-term recovery of motor function after stroke is a major public health issue and a big problem for patients and their families. But treatment provision is not satisfactory, research in this area is limited, and (at least some) patients feel abandoned by the health care system. A deeper understanding of the complexities involved in motor control and their interaction with the mechanisms of brain plasticity as well as psychological aspects of recovery is needed not only to maximize the treatment outcome for patients but also to tailor health service provisions and support infrastructures accordingly. Despite the incredible advancements in brain imaging and rehabilitation research, and the growth of knowledge on brain plasticity over the last 20 years, there is little we can offer to patients with minimal recovery at present. A targeted and interdisciplinary research effort is required to meet the need for research and treatment development. This is necessary for the sake of the individuals affected as well as those who fund the health and welfare systems.
